# Supplementary material for: Monitoring audience engagement using electrodermal activity during an inaugural lecture
Source: PLoS One. 2025 Jun 12;20(6):e0326091. doi: 10.1371/journal.pone.0326091 (PMC12161590; doi:10.1371/journal.pone.0326091)
Supplement: Attachment — Submitted filename: Response to reviewers.docx [file pone.0326091.s002.docx]

**Editor: The reviewers highlighted the need for greater clarity in defining participant groups and the methodology, The novelty of the study should also be better claimed, distinguishing between attentional engagement and interpersonal connection. Additionally, the manuscript should improve the discussion on the relationship between physiological synchrony and self-reported engagement. Please prepare a major revision accordingly, following the reviewers’ detailed comments.**

***Dear Dr. Greco,***

***We would like to thank you and the reviewers for your work and feedback on our mansucript. It helped us to improve the manuscript considerably. Below are the detailed point-by-point responses to the reviewers, but overall, the most important changes are:***

- ***Clarification of the added value of the current work in the Introduction and Discussion.***
- ***Clarification of the methods and terminology used.***
- ***Clarifying the rationale of the participants groups – we explain that this classification was used to operationalize expected differences in attentional engagement (in this case, due to the stronger or weaker personal connection with the speaker).***
- ***We discuss the relation between self-report and physiological synchrony more elaborately.***

***Furthermore, we obtained written permission from the people who can be recognized in the picture, to publish the picture.***

***On behalf of all authors,***

***Best regards,***

***Ivo Stuldreher***

**Reviewer #1:** Understanding audience engagement using physiological signals and synchronization is an interesting topic. However, I found the manuscript challenging to follow.

*We thank the reviewer for their thorough review of our manuscript. The review helped us to clarify the manuscript both concerning the methodology as well as the rationale and value of the study. Below, we respond to the individual comments separately.*

Comments:

• In the abstract, “investigated how well it distinguished between these groups of participants” is unclear because the groups were not previously defined. It seems the grouping is based on personal or professional relationships with the speaker, but this is not explicitly stated. Additionally, some participants could have both types of relationships.

*We clarify this now in the abstract, where we state:*

*“In this study, we moved out of the lab and monitored EDA of 30 individuals attending a real-life inaugural lecture. These individuals were labeled as belonging to either the personal or professional group, based on their relation with the speaker. We expected these groups to differ in their attentional engagement.”*

*The reviewer is right that the labelling is somewhat ambiguous. We discuss in the section ‘Distinguishing between participant categories’ in the Discussion that a number of participants with a professional relation to the speaker was also personally connected, which may be a reason why members of this group were harder to classify into the correct group than members of the personal group.*

• The statements “We compare the outcomes to results” and “Unlike analyses using individual’s EDA, analyses with inter-subject correlations do not require contextual event information” are unclear and need clarification.

*We rephrased the first statement in the abstract as follows:*

*“Where possible, we compare physiological synchrony results to results based on individuals’ EDA.“*

*We removed the second phrase.*

• It seems that the main finding is that EDA synchrony is not effective at detecting attentional engagement but is better at capturing a kind of “empathy” or relationship with the speaker. I suggest reflecting this more consistently throughout the paper, including the title. The results for engagement detection appear to be secondary in the analysis and are also not very positive.

*We do not think that synchrony in EDA directly captures empathy or the relationship with the speaker but that attentional engagement is the mediator. Earlier research (e.g. Stuldreher et al., 2020a; 2020b) indicates that EDA synchrony varies as a function of attentional instruction (i.e. what exactly in an audio presentation a participant needs to attend to), and that EDA synchrony marks engaging events (emotional sounds). Therefore, we expected that physiological synchrony might differentiate between individuals that are expected to differ in attentional engagement for a ‘natural’ reason (i.e., not instructed). The ‘natural’ reason that we chose is relationship with the speaker (and professional interest) - we expected that individuals that differ in their relationship to the speaker also differ in patterns of attentional engagement throughout the lecture. They are expected to experience the same lecture in different ways. Indeed, we find differences in EDA synchrony between individuals that differ in their relation to the speaker.*

*We now explain the known relation between physiological synchrony and attentional engagement, and the rationale of the study, more clearly in the Introduction.*

*Stuldreher, I. V., Thammasan, N., van Erp, J. B., & Brouwer, A. M. (2020a). Physiological synchrony in EEG, electrodermal activity and heart rate reflects shared selective auditory attention. Journal of Neural Engineering, 17(4), 046028.*

*Stuldreher, I. V., Thammasan, N., Van Erp, J. B., & Brouwer, A. M. (2020b). Physiological synchrony in EEG, electrodermal activity and heart rate detects attentionally relevant events in time. Frontiers in Neuroscience, 14, 575521.*

• Have the authors considered analyzing the correlation of the tonic component of EDA?

*In the current work we have not analyzed the correlation of the tonic component of EDA. The phasic component is considered more responsive to external events. As we hypothesize that synchrony in EDA is mainly driven by synchronized, consecutive skin conductance responses due to events, we focused on the phasic response only.*

• Section 2.6.2 suggests that the correlation of the SCRs (a more accurate term would be “phasic component”) was computed. However, subsequent sections about correlation analysis refer to participants’ EDA. Were raw EDA and phasic EDA correlations computed? Please clarify this.

*We are sorry for this confusion. Indeed, we computed correlations in the phasic component of EDA only. For simplicity we referred to synchrony in the phasic component as ISC-EDA, whereas we referred to the individual’s phasic EDA as SCR. This is indeed confusing, so we now consistently refer to individual’s phasic EDA as phasic EDA and to the correlations in phasic EDA as ISC-EDA.*

• I question the practical use of a system designed to capture the type of relationship (personal or professional) an audience member has with the speaker. It is uncommon to give a lecture primarily to family and friends. Please elaborate on the potential applications of this system.

*People can hear and see the same thing, but experience something entirely different. This is what we want to get at, and what we want to quantify. In this study, we used the (approximate) distinction of the type of relationship with the speaker (and professional interest) as a proxy of groups that we assume to differ in attentional engagement. We examine whether we can indeed distinguish between the two based on EDA. A potential practical application may be in the direction of tailoring and evaluating (informative) movies to an audience differing in expertise and interest.*

*We added this in the first sentences of the abstract and throughout the Introduction and Discussion.*

• I have not used EdaMove 4, but the statement about it being a “high quality” wearable seems unnecessary and unsupported by the data presented.

*We had originally added this, since EdaMove 4 can be considered as devices in between lab equipment (where the participant cannot move around) and a very rugged wearable that can be worn day and night (like a wristband). However, we now realize that does not come across like we intended, and the description of the equipment as well as its depiction in Figure 1 already convey our intended message. We thus removed the ‘high-quality’ statement from the text.*

• ISC-EDA was never defined. Initially, I thought ISC referred to the correlation, but since it was computed using the phasic component, it was labeled SCR. However, these terms appear to differ. It is unclear what SCR represents in the text, tables, and figures, as it does not seem to correspond to the phasic component of EDA for an individual.

*See also our response above. We removed the term SCR for the phasic component. Instead, we now use phasic EDA to refer to the phasic component of EDA and we use ISC-EDA to refer to the inter-subject correlations in the phasic component of EDA. We now introduce the term ISC-EDA in Section 2.6.2.*

• I initially thought the correlation was computed between the speaker and the participants, but it seems to be inter-subject. Furthermore, it appears that the correlation was performed within-group (same group) and between-group (other group). However, these groupings were not defined in the text and are not intuitive. This aspect of the manuscript was particularly difficult to follow.

*We now explicitly steer away from the suggestion that we study correlation between speaker and participants in the fourth paragraph of the introduction, and hope it is more clear by other clarifications of the methodology and motivation.*

• The statement “when we refer to inter-subject correlations, we refer to this participant-to-group metric” is confusing because the terms are used later in the text as if they represent different concepts: “The inter-subject correlations and participant-to-group inter-subject correlations.” The manuscript, especially the methodology, needs significant improvement to provide greater clarity.

*We removed the term participant-to-group correlation everywhere in the text after the statement, and clarified the methodology at several points.*

**Reviewer #2:** This study investigates physiological synchrony in electrodermal activity (EDA) as a real-time marker of attentional engagement in a real-life lecture setting. Results show that both physiological synchrony and individual EDA can differentiate between participant groups and detect engaging events but do not reliably align with self-reported engagement.
The study is overall well-executed and the analyses are comprehensive and detailed. Nevertheless, I believe that the novelty and contribution to the field may be not sufficient for a publication (as detailed below). A more detailed framing highlighting the novelty of this study may be useful to reach publication standards.

*We thank the reviewer for their thorough review of our manuscript. The review helped us to clarify the manuscript both concerning the methodology as well as the rationale and value of the study. Below, we respond to the individual comments separately.*

INTRODUCTION:
- MAJOR: The Introduction could be further expanded and elaborated.

*We expanded and elaborated the introduction by adding more background on the relation between physiological synchrony and attentional engagement, and highlighting the novelty (see below).*

- MAJOR: The overall novelty of this study should be highlighted more, as (at the current state) it does not seem to bring a significant contribution to the field.

We think the study is significant for the following reasons:

- We provide evidence that EDA as recorded in real-life conditions is informative on attentional engagement, where at the same time, factors such as low-level sensory effects, movement and speech cannot be the explanatory factor
- This is done for multiple aspects: detecting engaging events and distinguishing between groups that are expected to differ in attentional engagement
- The ground truth of the above (events and groups) is determined a priori and independent of self-report; our results on relating the measures with post-hoc self-report indicate that self-report is not always reliable and that EDA measures are complementary
- Different EDA metrics are examined: ISC-EDA which is a relatively novel marker that we put on the foreground, as well as more traditional phasic EDA and event-locked versions – comparing these gives us an idea of the sensitivity of the markers (both for cases when information about events is available, and event-related measures are an option; and when this is not the case)

This has now been processed in the introduction and the discussion.

- MAJOR: A general aim should be presented before specific research questions.

*We now present a general aim before presenting specific research questions:*

*“Our general aim is to identify whether interpersonal synchrony in EDA can be a marker of attentional engagement in a real-life setting, here an inaugural lecture.”*

- MINOR: Please clarify the role and meaning of peripheral measures, before explaining how they actually relate to attentional engagement.

*Yes, we do that now.*

METHODS:
- MINOR: Is there any particular reasons why the Bluetooth connection of the majority of heart rate sensors failed and not EDA sensors? This could be useful for future research as well.

*The EdaMove 4 sensors do not stream EDA data over Bluetooth. Instead, data is saved locally on the device itself. This differs from the heart rate sensors, that did not save the data locally, but streamed data over Bluetooth to a central computer. We now clarified this in Section 2.2. of the manuscript.*

- MAJOR: The event "pictures of Radboud colleagues" is used in self-report questionnaires but is not reported in events of interest. Could you specify the timing of this event? Was it used during the signal analysis? The analysis in 2.6.5 should be repeated on this event expecting opposite results compared to the brother's picture.

*While for the personal group, all participants knew the speaker’s brother, a similar case did not hold for the professional group and the pictures of Radboud colleagues. It turned out that only 6 of participants in the professional group were affiliated with Radboud, and of them, only 1 to 3 knew the depicted people well. While all participants were asked to rate this event, we therefore left out this event in further analysis. To prevent confusion, we now do not mention it at all.*

- MINOR: In Procedure: "attending professors leaving the room" -> "left"

*Thank you, we adjusted that phrase.*

- MAJOR: Since the EDA signal is often varying in time trajectory according to individual differences, what do the authors think about using lag-based measures, such as cross-correlation?

*This is a good point. We are aware of the interindividual differences in EDA responsiveness and varying time trajectories. However, implementing lag-based analysis such as cross correlation brings some challenges. For each individual one would have to compute the lag corresponding to the highest cross-correlation with all other individuals. In addition, the lag may not be constant, but vary in time as well. There thus are a lot of extra parameters compared to our simple linear correlations. As we are also considering potential real-time use, we preferred the ‘simple’ correlations over lag-based cross-correlations with varying lag between participants. In our previous work non-lag correlations proved to be successful.*

- MINOR: The acronym "ISC-EDA" is never explicitly defined in the main text.

*Indeed, we do that now. We also altered terminology slightly to avoid confusion. We refer to the phasic electrodermal activity no longer as SCR but as phasic EDA. We refer to inter-subject correlations in this phasic EDA as ISC-EDA consistently.*

- MINOR: Please define more details in the classification approach described at the end of paragraph 2.6.5.

*We elaborated this part:*

*“Furthermore, we determined a value for classification accuracy as follows. A best guess of which participants belong to the personal group on the basis on responses, would be done by picking the the N_personal_ participants with the highest response amplitudes to the picture, where N_personal_ corresponds to the number of participants with a personal relationship to the speaker (i.e., 14). The percentage of these 14 participants with the highest response amplitudes that indeed belong to the personal group is then the classification accuracy. The abovementioned approach was performed for ISC-EDA and phasic EDA.”*

- MINOR: 2.6.6; also this analysis should be clarified.

*We elaborated on it so that the reader will at least get the gist without having to read the provided reference:*

*“We marked the ISC-EDA and EDA traces with time intervals during which the engaging events took place. During these times, an event detection would thus be considered correct (True) and an event detection during other moments would be considered incorrect (False). The moments in time where the trace is higher than a threshold t are marked as an event (Positive) and the moments in time where the trace is lower than t are marked as a non-event (Negative). Rather than using a single value for t, we use a gradually changing threshold t, ranging from the minimum to the maximum observed value of the trace. For each iteration of t, we can then define the true positives (TP), false positives (FP), true negatives (TN) and false negatives (FN). Using this, the true-positive rate or sensitivity (TPR) and the false-positive rate (FPR) or specificity can be computed. Plotting TPR against FPR provides the receiver operating curve (ROC). Detection performance was assessed using the standard metric of the area under the ROC (AUC of ROC).”*

- MINOR: Analyses of self-report questionnaires are not specified in the Methods.

*Indeed, this has now been added (Section 2.6.7)*

- MINOR: A public version of the dataset should be made available, at least for the Reviewers (there is a specific option in OSF). Until now, it was possible to access it only as contributor.

*Sorry, we were unaware the repository was still private. The OSF repository has now been made publicly accessible.*

RESULTS:
- MINOR: "highest physiological synchrony with yields 69%" -> "which"
- MINOR: "Figure 2 shows that participants that have a personal relationship with the speaker have significantly..." is actually Figure 3.

*Thank you for noticing these two points, we corrected these.*

DISCUSSION:
- MAJOR: Please discuss the absence of a relation between physiological synchrony and self-report measures in light of Gashi et al.'s [12] results.

*We corrected our description of Gashi et al.’s results in the introduction. They actually did not find that synchrony between presenter and audience member predicted self-reported levels of engagement, but that it predicted the agreement between presenter and audience member on self-reported levels of engagement. In fact, while their study differed from ours (e.g. focusing on synchrony between speaker and audience, rather than between audience members; removing almost half of the data due to low data quality; using different analyses) their results are in line with ours such that synchrony did not correlate with self-reported engagement. Gashi et al. argue that more data is required to establish such a connection which we agree on. This is now discussed in the section on self-reported engagement scores in the discussion.*

- MAJOR: most results (except for the event detection) are about the SCR and not ISC-EDA. Showing that the phasic component of EDA is related to attentional engagement is not an overwhelming result, so the novelty of this study should be highlighted more clearly.

ISC-EDA is the more novel marker, and our starting point. For some questions such as distinguishing between groups that are expected to differ in attentional engagement, we cannot rely on phasic individual EDA (without specific information about crucially distinguishing events) and we demonstrate that ISC-EDA is informative. For other questions (detecting relevant events), we here tested the sensitivity of ISC-EDA relative to phasic EDA, and found that ISC-EDA is not surpassing the arguably simpler measure of phasic EDA, which is a valuable result.

Aside from this, and as alluded to in our second response above, we think that also from the phasic EDA perspective only, our study significantly adds to previous literature. Studies that associate real life events with EDA and interpret results in terms of arousal normally cannot dissociate between effect of sensory stimulation and/or body movement from purely cognitive or emotional factors. We need to keep in mind that sensory stimulation and/or body movement are likely also related to cognition and emotion, and can therefore not be disentangled using self-report or performance measures. In our real-life study, audience members sit still and experience the same stimuli. Still, EDA (as reflected in ISC-EDA and phasic EDA) depends on group (personal or professional relationship with the speaker) as we had hypothesized because of the expected difference in attentional engagement pattern between these groups.

- MINOR: Fig. 4, low panels: adding a boxplot or violin plot to the points could be useful to visualize the results.

*We included a violin plot in the lower panels of Fig 4.*
